# Behavioral and Psychological Outcomes Associated with Skin Cancer Genetic Testing in Albuquerque Primary Care

**DOI:** 10.3390/cancers13164053

**Published:** 2021-08-12

**Authors:** Jennifer L. Hay, Kimberly A. Kaphingst, David Buller, Elizabeth Schofield, Kirsten Meyer White, Andrew Sussman, Dolores Guest, Yvonne T. Dailey, Erika Robers, Matthew R. Schwartz, Yuelin Li, Keith Hunley, Marianne Berwick

**Affiliations:** 1 Department of Psychiatry and Behavioral Sciences, Memorial Sloan Kettering Cancer Center, New York, NY 10065, USA; schofiee@mskcc.org (E.S.); liy12@mskcc.org (Y.L.); 2 Cancer Communication Research, Huntsman Cancer Center, University of Utah, Salt Lake City, UT 84112, USA; kim.kaphingst@hci.utah.edu; 3 Klein Buendel, Inc., Golden, CO 80401, USA; dbuller@kleinbuendel.com; 4 New Mexico VA Health System, Veterans Health Administration, Albuquerque, NM 87108, USA; kirsten.white@va.gov; 5 Department of Family and Community Medicine, University of New Mexico, Albuquerque, NM 87102, USA; asussman@salud.unm.edu; 6 University of New Mexico Comprehensive Cancer Center, Albuquerque, NM 87102, USA; dguest@salud.unm.edu (D.G.); ytala1352@gmail.com (Y.T.D.); Erika.Robers@gmail.com (E.R.); MSchwar@salud.unm.edu (M.R.S.); 7 Department of Anthropology, University of New Mexico, Albuquerque, NM 87102, USA; khunley@unm.edu; 8 Department of Internal Medicine, University of New Mexico, Albuquerque, NM 87102, USA; MBerwick@salud.unm.edu; 9 Department of Dermatology, University of New Mexico, Albuquerque, NM 87102, USA

**Keywords:** genetic testing, sun protection, primary care, Hispanics, skin cancer risk

## Abstract

**Simple Summary:**

Genetic information is publicly available but research examining the best use of such information has not engaged diverse members of the public. We examined public reactions to melanoma genetic testing (using the melanocortin-1 receptor [*MC1R*] gene) in a study randomizing (like the flip of a coin) 600 diverse primary care patients to a *MC1R* test offer or usual care. We found that testing did not improve sun protection and skin cancer screening, nor did it lead worry to increase. However, groups less aware of their skin cancer risk, including those who thought their risk was “unlikely” at the start of the study, showed significant improvements in sun protection at three months. In conclusion, testing might be very helpful for certain people who have the most to learn about their risk, who may become motivated to protect themselves from the damaging effects of the sun as a result of skin cancer genetic testing.

**Abstract:**

Public availability of genetic information is increasing; thus, efforts to improve diversity in basic and translational research in genomics is a top priority. Given the increasing U.S. incidence and mortality of melanoma, and the prevalence of common melanocortin-1 receptor (*MC1R)* gene melanoma risk variants in the general population, we examined genomic testing of *MC1R* for skin cancer risk in a randomized controlled trial in Albuquerque, New Mexico primary care. Participants were 48% Hispanic and were randomized 5:1 to a *MC1R* test invitation or usual care. We assessed 3 month sun protection, skin cancer screening, and skin cancer worry outcomes associated with testing, and key effect moderators (e.g., cancer risk perceptions, and skin cancer risk factors). Our findings indicate that the primary outcomes were unchanged by the *MC1R* test offer, test acceptance, and level of risk feedback. Moderator analyses showed that those with lower risk perception, and those with skin that readily tans, significantly increased their sun protection in response to higher than average risk feedback. Risk feedback did not prompt cancer worry, and average risk feedback did not erode existing sun protection. This study paves the way for the development of tailored strategies to address low skin cancer risk awareness in this understudied context of public health genomics.

## 1. Introduction

Genetic information regarding health risk, and cancer risk specifically, is available through multiple sources—both within and outside of the U.S. medical and public health systems, and there are relatively high levels of public awareness concerning this availability [1]. Genetic information about cancer risk is of interest to the general population, and this interest stems from a range of motives, including personal curiosity, empowerment regarding health, efforts to enhance cancer prevention and control, as well as to enhance family awareness and cancer risk reduction [1]. Direct-to-consumer (DTC) genetic testing has been available for several decades. By 2021, it is estimated that more genetic testing will be conducted outside than inside the traditional health system [2,3]. Genetic information provided by DTC channels is controversial in the medical community, constituting criticisms regarding test validity, utility and comprehensibility of results, as well as the potential to increase worry and poor outreach to diverse populations [4]. Nonetheless, 40% of the population is aware of DTC testing [5,6], and utilization has expanded accordingly [7]. As of 2018, the consumer genomics industry estimated that 12+ million individuals had submitted samples for DTC genetic testing. By early 2019, an estimated 26 million people contributed their genetic results to one of the large consumer genomics databases (e.g., AncestryDNA and 23andMe) [2]. NIH also is collecting biospecimens on one million members of the population, targeting recruitment to racial and ethnic minorities in order to further understanding of gene–environment interactions and the ideal return of results through the All of Us Research Project (https://allofus.nih.gov/) (accessed: 26 July 2021). Costs for sequencing have been decreasing rapidly [8]. Despite the scale achieved for DTC genetic testing, the health promotion opportunities, particularly in diverse populations [9], that are afforded by this momentum have not yet been realized. Although members of racial and ethnic minority groups stand to benefit from genomic technologies, to date, those who pursue personalized genetic information have been disproportionately non-Hispanic White and at higher socioeconomic, education, and literacy levels [10]. Additionally, DTC testing has largely been conducted outside the healthcare system; the potential for some integration into established healthcare could improve access for underserved populations, and present enhanced opportunities for links between genetic test result feedback and counseling regarding health promotion and maintenance.

Melanoma is the 5th most common cancer in the U.S., and its incidence has tripled in the last four decades _ENREF_1 [11,12,13,14,15]. Furthermore, over 4.3 million adults are treated for keratinocyte cancers (i.e., squamous cell carcinoma (SCC) and basal cell carcinoma (BCC)) annually in the U.S. [16], and incidence is increasing steadily [17,18]. Despite low associated mortality, keratinocyte cancers are common and expensive to treat [19]. _ENREF_23 Most melanoma is caused by ultraviolet radiation (UVR), largely from sun exposure [20,21,22,23]. Limiting UVR exposure is estimated to substantially reduce melanoma rates [20]. Keratinocyte cancers share UVR exposure as a major risk factor [24,25].

The melanocortin-1 receptor (*MC1R)* gene encodes a protein that signals the production of melanins. *MC1R* exhibits great genetic variability, and a large proportion of individuals of European ancestry, including Hispanics, carry at least one *MC1R* risk variant [26,27,28]. The inheritance of specific *MC1R* variants is a robust marker of increased risk of melanoma and keratinocyte skin cancers [26,29,30,31]. Associations between *MC1R* variants and skin cancer are stronger in individuals with “sun-resistant” phenotypic characteristics (e.g., darker hair, scant freckling) than in those with “sun-sensitive” characteristics (e.g., red hair, easy burning, freckling) [26,27]. The theoretical reduction of melanomas through the “removal” of one of the nine most prevalent *MC1R* variants (i.e., attributable risk) ranges from 1.2 to 8.9% and approaches 40% when summed across variants [26]; this proportion is 30% and 45% for basal and squamous cell skin cancers, respectively [31]. Variants tested for, and accession numbers, include the following: V60L (rs1805005), D84E (rs1805006), V92M (rs2228479), R142H (rs11547464), R151C (rs1805007), I155T (rs1110400), R160W (rs1805008), R163Q (rs885479) and D294H (rs1805009). As such, the *MC1R* genotype conveys information about inherited skin cancer risk for a broad spectrum of individuals, including Hispanics. Most (50–70%) of the general population of European ancestry and 50% of Hispanics have at least one risk variant at *MC1R* [26,28], even in individuals with darker complexions [32,33], making genetic testing for *MC1R* variants a potential novel route to motivate sun protection and skin cancer screening in the general population. In New Mexico, specifically, those who identify as Hispanic derive from mating between Native Americans (resident in the region for at least 13,000 years) and Europeans and Africans, who arrived over the past 400 years. Recent studies indicate that the majority of ancestry is European (mean = 71%), followed by Native American (27%) and African (2%) [34,35,36]. Given the documented frequency of *MC1R* variants in Europe (26–28), many New Mexican Hispanics today carry *MC1R* variants.

Genomic testing of *MC1R* for skin cancer risk may promote behavior change across ethnically and racially diverse populations [37], especially among Hispanics, who generally have low skin cancer risk awareness [38,39,40,41,42,43]. Meta-analytic work has shown that genetic information motivates behavior change and does not generate high levels of distress or worry [44], yet the use and impact of genetic information in diverse community settings [45] is extremely understudied [46]. This randomized controlled trial (“SOMBRA: Skin Health Online for Melanoma: Better Risk Assessment”) was conducted in a diverse primary care population in Albuquerque, New Mexico, which experiences year-round sun exposure [47]. We examined 3 month sun protection, skin cancer screening, and skin cancer worry outcomes associated with *MC1R* test offer and completion. Our research questions were as follows. First, what are the 3 month outcomes (i.e., overall sun protection, skin cancer screening, cancer worry) associated with the receipt of higher (compared to average) risk *MC1R* feedback? We also examined outcomes associated with receiving a *MC1R* test offer (compared to usual care) and accepting that offer (compared to declining the offer). Second, were there significant moderators of the relationship between the receipt of higher vs. average genetic risk findings and the 3 month outcomes (i.e., overall sun protection, skin cancer screening, cancer worry)? Potential moderators included participant health literacy, perceived importance of genetic information, cancer risk perceptions, demographics, and skin cancer risk factors, all of which may influence behavioral and psychosocial outcomes associated with skin cancer genetic testing [48,49,50,51,52,53,54,55].

## 2. Materials and Methods

### 2.1. Participants

Bilingual Project Assistants (PAs) approached primary care patients in University of New Mexico (UNM) outpatient primary care clinics with SOMBRA study invitation flyers (English and Spanish) and National Cancer Institute skin cancer information for diverse skin types (available in English and Spanish versions: “Anyone can get skin cancer”) [56]. Patients were eligible if they were registered in any UNM clinic for at least six months, assigned a UNM primary care provider, were aged 18 years or older, and fluent in English or Spanish. All study procedures and materials were approved by the University of New Mexico (UNM) Institutional Review Board (ClinicalTrials.gov identifier: NCT03130569).

### 2.2. Procedure

**Figure 1:**
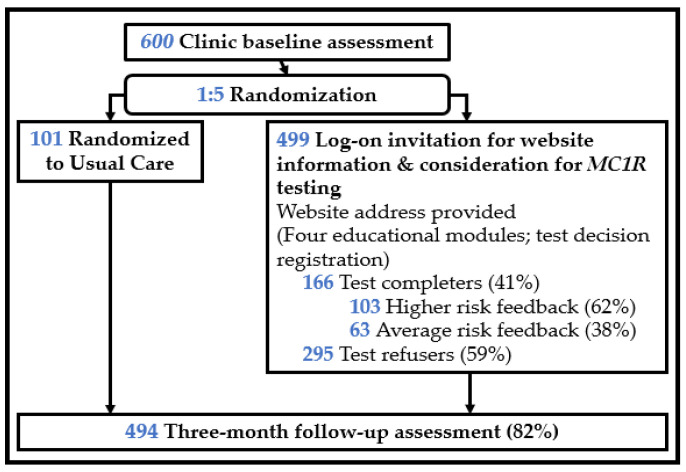
Study design. Note: of the 499 who received a log-on invitation, 37 requested but did not return a kit, and 1 returned a kit but no risk level was recorded.

Patients who were eligible completed a one-minute screening survey that assessed perceived importance in learning about genes and health risks, skin cancer risk perceptions, and demographics. Refusers were asked their reasons for study refusal. Those willing to participate completed the informed consent and baseline assessment and were then randomized 5:1 to an invitation to consider *MC1R* testing or usual care (control group), balanced across Hispanic vs. non-Hispanic ethnicities. Overall, 600 participants enrolled in the RCT and completed the baseline assessment; 499 were randomized to the *MC1R* testing offer; and 101 were randomized to usual care. Usual care participants did not receive an invitation to log on to the study website. All participants received National Cancer Institute skin cancer information brochures about skin cancer risk in diverse skin types (available in both English and Spanish) [47]. See Figure 1 for study design.

Those randomized to the intervention arm were invited to log onto the study website [47] to read the three educational modules regarding *MC1R* testing and then register their decision to participate in testing. Patients without internet access were offered the opportunity to view the website via paper format. Participants who chose testing were mailed a kit to obtain their DNA, and if they so desired, provided a saliva sample for genetic testing. Those participants who sent in their sample received a report with their results in the form of genetic risk feedback stating that they were at either average risk, based on the presence of no *MC1R* variants associated with melanoma risk, or higher risk, based on the presence of at least one *MC1R* variant associated with risk of developing melanoma [55]. The design of this risk feedback, using plain language and clear communication guidelines, was adapted from the Multiplex Initiative study [57]. Participants who opted for testing received a follow-up telephone call two weeks after receipt of their results, for an assessment of comprehension, satisfaction with risk information, and distress level; these findings are reported elsewhere [55]. Primary outcomes were assessed at three months. Those participants randomized to usual care were offered *MC1R* testing as a courtesy after completion of the final 3 month follow-up outcome assessment, and 57 of 101 usual care participants requested it.

### 2.3. Measures

#### 2.3.1. Outcome Measures

Sun protection: The frequency of use of five sun protective behaviors while in the sun—sunscreen, shade-seeking, hats, protective clothing, and sunglasses—were reported at baseline and again at the 3 month follow-up. Participants rated each behavior on a 5-point frequency scale ranging from 1 = Never to 5 = Always [58]. We calculated overall sun protection (summary score including all 5 sun protection strategies ranged from 5 to 25) as a primary outcome variable.

Skin cancer screening: At the baseline assessment, we assessed whether participants had ever had their skin checked for skin cancer (from head to toe) by a healthcare professional. At the 3 month follow-up, they were asked whether they had received skin cancer screening since they had started the study.

Cancer worry: Worry items were drawn from Lerman and colleagues [59] on 4-point scales, including the frequency of worry about the possibility of getting skin cancer in the past two weeks (1 = rarely/never to 4 = all the time), and the level of concern about the possibility of developing skin cancer (1 = not at all to 4 = very concerned); a summary score ranged from 2 to 8.

#### 2.3.2. Predictors

We examined three predictors. First, we explored the outcomes associated with the receipt of an *MC1R* test offer (vs. usual care). Second, among those who received the test offer, we examined the outcomes associated with accepting that offer (vs. declining that offer). Test offer acceptance was defined as logging on to the website and requesting a test kit. Declining the test offer included those who actively declined the test offer on the study website, as well as those who did not log on to the website at all. Third, of those who completed testing by accepting the test offer, we tested the outcomes associated with the receipt of higher (vs. average) risk test results. See Figure 1.

#### 2.3.3. Moderators

We included participant health literacy, perceived importance of genetic information, cancer risk perceptions, demographics, and skin cancer risk factors in the models as potential effect moderators. Health literacy was assessed with three items: (1) level of confidence in filling out medical forms independently, (2) frequency of needed assistance reading hospital materials, and (3) frequency of problems learning about medical conditions because of difficulty reading hospital materials (1 = not at all/none of the time to 5 = extremely/all the time). These items have _ENREF_133 good sensitivity across diverse literacy levels [60,61]. Moderators were assessed through the use of interaction terms, using standardized effect sizes, rather than the use of *p*-values for hypotheses testing, to avoid the problem of multiple comparisons.

Perceived importance in learning about ones’ genes and associated health risks was assessed (1 = not at all important to 7 = very important) [53]. Perceived skin cancer risk was assessed using three widely used scales [62,63], including two absolute verbal likelihood scales (1 = no chance to 7 = certain to happen; and 1 = unlikely or 2 = likely to happen) with “don’t know” response options [64]. A comparative likelihood assessment (1 = well below average to 5 = well above average, compared to individuals of participants’ age and sex) was also included. Demographics included ethnicity (participants were asked if they were of Hispanic, Latino/a, or Spanish origin), race, sex, educational attainment, age, and income. Skin cancer risk factors included history of cancer, family history of skin cancer, skin type (burnability, tannability), and intermittent sun exposure (lifetime number of sunburns) [65,66].

### 2.4. Biostatistical Approach

Sun protection and cancer worry were treated as continuous 3 month outcomes, and skin cancer screening as a dichotomous outcome. Attrition between the baseline and 3 month follow-up was described; completers were compared to non-completers based on baseline characteristics. Differences by attrition were tested via a series of independent sample *t*-tests and Chi-square tests, for continuous and categorical variables, respectively. The general framework for analysis was generalized regression modeling, regressing 3 month outcomes on predictors (i.e., intervention arm, test acceptance, and risk results) and adjusting for baseline. For our first research question, the 3 month outcomes were compared across *MC1R* test offer (compared to usual care), test acceptance (compared to declined), and receipt of higher (compared to average risk results). Models were fitted both with and without adjustment for baseline characteristics that differed based on attrition status. For our second research question, another series of regression models were used to assess moderator effects with the receipt of higher, compared to average, risk test results on 3 month outcomes, separately. For these models, the baseline outcome, potential moderator (e.g., participant health literacy), test results, and interaction of moderator and test results were included. Eta-squared (η^2^) in a regression model is the ratio of the variance explained by a parameter of interest. The interaction term was the parameter of interest, with η^2^ reported as a measure of standardized effect sizes for continuous outcomes and standardized odds ratios for the binary skin exam outcome. This strategy of using standardized effect sizes and then probing non-trivial associations, rather than merely relying on *p*-values, reduced the risk of inflated family-wise error rate when investigating a large number of potential moderators [67]. For η^2^ in multiple regression, values of 0.02, 0.13, and 0.26 were considered small, medium, and large effect sizes, respectively. For interaction odds ratios (iOR) with standardized variables and rare outcomes, values of 1.68, 3.47, and 6.71 were considered small, medium, and large effects [68]. Significant findings were investigated with stratified analyses. All statistical analyses were conducted in SAS version 9.4.

## 3. Results

As reported previously [69], 1998 primary care patients were approached, 917 (46%) agreed to be screened for eligibility, and of the 726 patients who were eligible for the study, 621 (86%) consented to study participation; 21 did not complete the baseline assessment. Study acceptance was higher in non-Hispanic whites compared to Hispanics, and those with higher (>high school) compared to lower educational attainment (<high school) but did not differ on other demographic factors [69]. Further, those with higher perceived skin cancer risk and those with greater interest in learning about genes were more likely to participate. Participants who answered “don’t know” to absolute perceived skin cancer risk were *less* likely to participate in the study than patients who gave another scale response; these psychosocial factors remained significant predictors of study participation after adjustment for ethnicity and education [54].

Of the 600 study participants, 79% were female, 48% Hispanic, 71% white, and 77% had more than a high school education. Most (*n* = 494, 82%) completed a 3 month follow-up. Participants who were not retained at the 3 month follow-up were more likely to be Hispanic (63% vs. 45%, *p* < 0.01), have lower educational attainment (*p* < 0.01) or income (*p* < 0.01), have no family history of cancer (79% vs. 63%, *p* < 0.01), have responded “don’t know” to absolute perceived risk (18% vs. 9%, *p* < 0.01), and have rated their risk as “unlikely” at baseline (65% vs. 48%, *p* = 0.02). Overall, those who completed the study were predominantly female (79%), white (72%), had at least some college education (80%), and were 53 years of age (mean = 53.2, SD = 14.0). Baseline levels of sun protection, skin screening, and cancer worry were unrelated to the study completion status. Baseline characteristics both overall and by study completion status are shown in Table 1 and Table 2.

At baseline, only one quarter (24%) of participants had sun protection scores less than 15 across the five behaviors; the mean score was 17 (on a 5 to 25 scale), which translates to somewhere between “sometimes” and “often.” However, only one third (37%) of the participants had ever received skin cancer screening, and skin cancer worry was extremely low (mean = 2.5, SD = 1.1 on a 2 to 8 scale).

Most participants reported little to no change in all outcomes between the baseline and 3 month follow-up. For example, the median sun protection change score was zero (inter-quartile range −2 to 2; possible range −20 to +20), and only 5% of participants had a change score of more than five points in either direction. Similarly, only 10% of participants who had never had skin screening received one in the study time period, and cancer worry scores increased slightly, by 0.03 points on average. In models adjusted for baseline outcomes only, the predictors (test offer, test completion, and test feedback level) were not significantly associated with sun protection, skin cancer screening, or cancer worry at 3 months (Table 3). The baseline scores were strong predictors for most of these 3 month outcome measures. Models adjusted for participant characteristics that varied by attrition (i.e., ethnicity, education, income, family skin cancer history, and perceived risk) showed results consistent with the pattern outlined above.

Most moderators had no significant interaction effects with level of *MC1R* risk feedback (higher vs. average risk) on outcomes; however, some of these effects stood apart with small but significant effect sizes. See Table 4.

**Figure 2:**
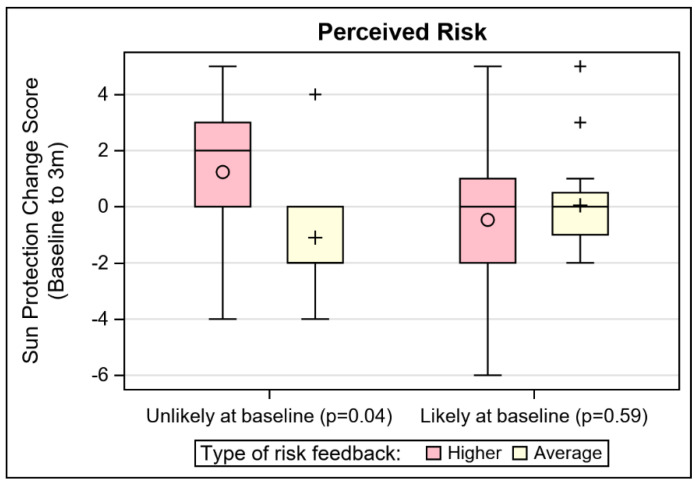

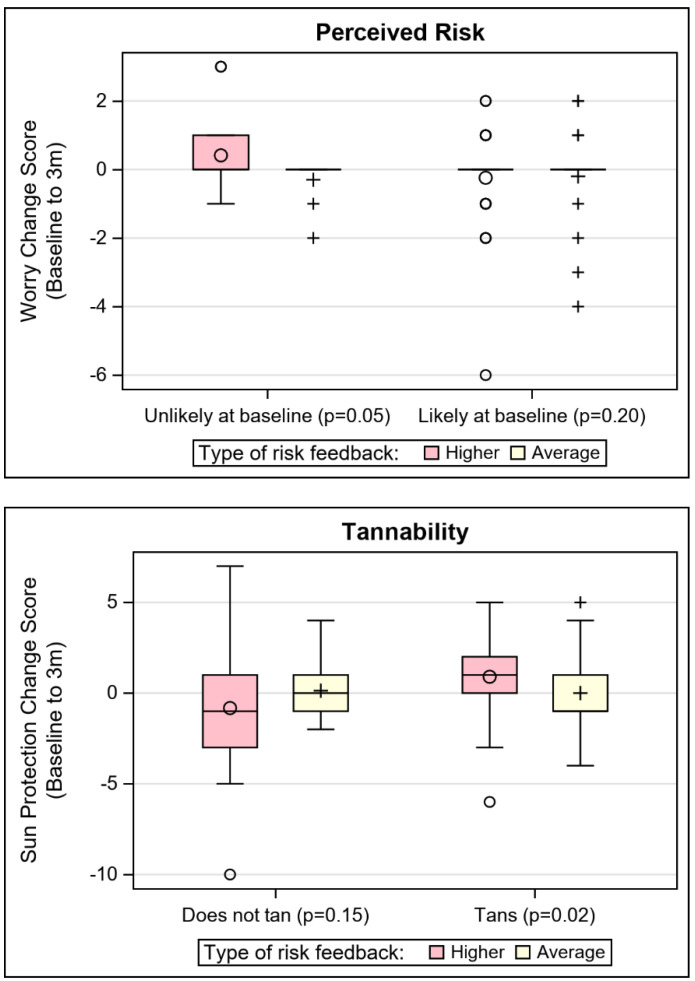
Depiction of notable sun protection and skin cancer worry moderator effects.

Tannability (η^2^ = 0.03; *p* = 0.01) and perceived risk (absolute, dichotomous; η^2^ = 0.02; *p* = 0.04) were significant moderators of the effect of risk feedback on sun protection. The same risk perception item had a non-trivial effect size (η^2^ = 0.05; *p* = 0.20) for moderation of risk feedback level on cancer worry. These moderations effects are depicted in Figure 2, where the distribution of change scores for sun protection are plotted by the risk feedback level and the moderators. For participants who reported that their skin does tan, those who received higher risk *MC1R* feedback reported significantly greater improvement in sun protection (*p* = 0.02) than those who received average risk MC1R feedback. For participants who believed they were unlikely to develop skin cancer at baseline, those with higher risk feedback had significantly greater improvement in sun protection (*p* = 0.04), compared to those who received average risk feedback. These associations did not hold for participants who reported that their skin did not tan or who reported at baseline a high likelihood of skin cancer risk. Though the stratified results for risk perception were not significant for cancer worry, the same pattern held (i.e., among those who believed that they were unlikely to develop skin cancer at baseline, those with higher risk feedback had greater increase in worry). Different patterns of moderators were seen for the effect of *MC1R* risk feedback level on the skin cancer screening outcome. Educational attainment, history of sunburn, and family skin cancer history stood apart as potential moderators after controlling for skin screening history, based on effect sizes. First, among those who had never received prior skin cancer screening, higher risk feedback was associated with skin cancer screening completion at 3 months among those with lower educational attainment (iOR = 8.32, *p* = 0.11). Cell sizes were too small to make similar inference for participants who had previously screened. Second, regardless of their skin cancer screening history, among those who had no history of sunburn, receipt of average risk results predicted 3 month skin cancer screening (iOR = 36.97, *p* = 0.01). Third, the family history effect was complex. Among those who had never been screened, only those with a family history of skin cancer and higher risk *MC1R*, or those with no family history of skin cancer and average risk *MC1R,* completed any screening at follow up. Among those who had never been screened, none of the participants who received only higher risk feedback, or had a family history of skin cancer, reported screening at the follow up time period (iOR = 16.65, *p* = 0.05).

## 4. Discussion

The provision of genetic feedback information to the general population is controversial, with concerns raised about the potential value of such feedback for health promotion and medical decision making, as well as whether such information might prompt health worry or distress. At the same time, the availability of genetic information to the general public has increased dramatically in recent years [3], so exploring the public reaction to such information is needed across diverse populations who have the potential to benefit from it [4]. A large proportion of individuals of European ancestry, including New Mexico Hispanics, carry at least one *MC1R* gene variant that confers increased melanoma risk [26,27,28], although recent evidence found that the risk conferred by such variants may differ by specific Hispanic populations [70]. Nonetheless, the inheritance of specific *MC1R* variants may be a robust marker of increased risk of melanoma and keratinocyte skin cancers [26,29,30,31]. In the current study, receiving higher (compared to average) risk feedback did not increase sun protection or skin cancer screening at three months. Provision of a test offer, and completion of testing, also did not influence subsequent sun protection. Given the concerns that genetic information provided by DTC channels may increase worry [4], our findings are promising in that worry outcomes were also not influenced by *MC1R* test offer, completion, risk feedback level. These findings are especially important, given the primary care environment utilized, with about half the sample identifying as Hispanic, a population that has received little attention in the context of DTC testing [71].

In our study population, sun protection utilization was already relatively high at baseline assessment, perhaps because Albuquerque has year-round sun exposure, which may serve to support sun protection habits over time. As such, the impact of offering *MC1R* testing, completing testing, and receiving risk feedback results may have been suppressed due to ceiling effects, creating reduced opportunity for behavioral impact. In previous analyses, participants who completed testing reported high levels of interest and engagement in their test feedback results and reported determination to improve sun protection behavior after receiving feedback, and such determination was significantly higher in those who received higher risk feedback, compared to average risk feedback two weeks after test receipt [55]. Those with a sunburn history were more likely to complete testing [69]. It may also be that there are elements of behavioral impact that were not measured in the current study that might ultimately contribute to the personal and health utility of *MC1R* feedback, such as longer-term behavioral habits, improved engagement and perceived control over sun protection choices. Fortunately, our findings indicated that receipt of average risk *MC1R* feedback did not lead to the reduction or elimination of sun protection. Caution has been raised about whether provision of average risk results may provide risk behavior justification [72]. These findings provide some solace that the provision of “normal” or “good news” genetic test results did not undermine protective behavior, consistent with prior work in highly educated samples [73]. Findings on the sun protection outcomes are inconsistent with meta-analytic evidence supporting the fact that genetic test findings positively impact motivation for health promotion [44], yet they nonetheless shape the important questions going forward in the area of genomics and behavior change, as well as precision approaches to cancer prevention that engage multiple types of personalized risk information, more broadly.

Based on moderation analyses examining interactions, our findings indicate some specific groups that may be particularly responsive to higher risk feedback in increasing their sun protection. These include adults with high skin tannability, and those who, at baseline, judged their skin cancer risk as “unlikely.” These individuals may have been unaware of their risk and may be more influenced by the motivational impact of the risk feedback; it may also be that unexpected or surprising results are more motivational, which is an important topic for further research. Theories of risk communication predict that messages that increase fear, such as high-risk *MC1R* test feedback, should motivate people to take precautions that they perceive will reduce their risk, such as sun protection [74,75]. Consistent with this, moderation analyses found that skin cancer worry was somewhat higher for those who judged their baseline skin cancer risk as “unlikely” and received higher versus average risk feedback. The risk communication theories predict that the increased surprise in learning of a higher risk produces discomfort that can be reduced by taking more sun safety precautions. Further work should examine both the psychosocial and behavioral outcomes of *MC1R* testing. As such, a subgroup approach, consistent with precision prevention efforts, may be a useful strategy for future research since some subgroups may benefit more from skin cancer genetic risk feedback than others.

Further, skin cancer worry was not increased by *MC1R* testing. This helps allay concerns [44] that the provision of such feedback may increase health worries, and/or distress, particularly in the absence of detailed genetic counseling, and in DTC testing of the general population outside of the high-risk setting. While the current study examined 3 month skin cancer worry outcomes, the findings are consistent with demonstrated short-term impact of testing; distress and negative effects were low, and satisfaction with testing and receipt of results were quite high two weeks after receiving results [55].

Future research should target subgroups of the general population who have the most to gain from skin cancer risk information that is highly personalized and may thus carry the most unique motivational potential. This may include selection for those at highest behavioral risk, such as those who engage in indoor or outdoor intentional tanning, do not use sun protection, have a history of multiple sunburns, or have low skin cancer risk awareness (e.g., low perceived risk; reports that they “don’t know” their risk). Further, selection for youth in secondary schools and universities to heighten awareness of skin cancer is a promising area for future research. This may include large portions of the general population who tan and/or do not use adequate sun protection [76]. In the current study, patients with low risk perceptions were significantly more likely to refuse study participation than those with higher risk perception [54], which may have decreased the ability to detect an overall impact of the genetic testing feedback. It is an important element of study rigor that we were able to well-characterize these study refusers. Strategies are needed to recruit populations with misplaced low risk perception who would likely benefit behaviorally from the receipt of higher-risk feedback. This finding highlights the importance of delineating the motives and attitudes of those who actively pursue testing versus those who decline it, as we did in the current study. It is also important to further specify whether such risk information must be paired with specific intervention content to enhance it, such as information to support the efficacy of sun protection to reduce risk and the ability of individuals to adequately practice sun protection over time, across diverse situations and contexts, to achieve these benefits [77].

The current study has limitations as well as strengths. First, we assessed primary care patients in only one area of the country, which already had a generally high prevalence of practicing sun protection, which reduces generalizability. Second, we relied on self-reported sun protection, although self-report has been found to be a valid indicator of objective sun protection usage [78]. Future work should confirm and expand on these findings using objective measures for UV exposure and objective sunburn, even though self-reports are appropriate for assessing cognitions, such as risk perceptions, the importance of genetic testing, and skin cancer worry. Third, given our study design and recruitment of participants, regardless of their interest in following through with *MC1R* testing, we had relatively small samples of those who received feedback. While this limited the statistical power to test moderation on risk feedback (higher versus average risk), our study design allowed for examination of behavioral and psychosocial outcomes critical to real-world use of skin cancer genetic testing feedback, where those who follow through with testing may differ significantly from those who do not. Future use of larger samples will also allow for further examination of moderators, such as differences in educational attainment. These important influences on behavior may not be measurable in study designs where all participants recruited are already interested in pursuing genetic testing. Our exceptionally high recruitment rates (86% of eligible primary care patients) were a study strength, along with the careful measurement of interest in, and completion of, testing; most (88%) who visited the website to consider testing went ahead and ordered a test kit [69]. Recruitment of only those who were already interested in and willing to undergo skin cancer genetic testing may have resulted in a much smaller, self-selected sample; as such, our rigorous approach closely matches the current real-world situation, where genetic technologies are available but voluntary.

## 5. Conclusions

Efforts to improve diversity in genomic research, both basic and translational, are a top priority [79]. The current study examined an understudied element of public health genomics [80], genetic testing for common susceptibility and behavioral and psychosocial outcomes in a diverse, primary care patient population. In particular, the study successfully engaged Hispanics in discussion and engagement with skin cancer genetic testing. We did not find main effects for *MC1R* testing feedback on behavioral outcomes, including sun protection and skin cancer screening, and similarly did not find any increase in skin cancer worry, an important and promising finding. However, we identified moderators with relatively large effect sizes, including low risk perceptions, which suggest targets for the deployment of tailored strategies, using skin cancer genetic testing in diverse subpopulations that may be most readily impacted by risk feedback.

## Figures and Tables

**Table 1:** Patient baseline characteristics and testing results by follow-up status (N = 600).

Ethnicity, Hispanic	All, *n* (%)	Study Completers, *n* (%)	Lost-to-Follow-Up, *n* (%)	*p*-Value
	286 (48%)	220 (45%)	66 (63%)	<0.01
Race				
American Indian or Alaskan Native	15 (3%)	12 (2%)	3 (3%)	0.11
Asian	12 (2%)	10 (2%)	2 (2%)	
Black or African American	15 (3%)	14 (3%)	1 (1%)	
Native Hawaiian or Pacific Islander	2 (0%)	1 (0%)	1 (1%)	
White	423 (71%)	357 (72%)	66 (62%)	
Other	132 (22%)	99 (20%)	33 (31%)	
Gender, female	473 (79%)	390 (79%)	83 (78%)	0.88
Education				
<HS	46 (8%)	28 (6%)	18 (17%)	<0.01
HS or GED	94 (16%)	66 (13%)	28 (26%)	
Some college	142 (24%)	121 (24%)	21 (20%)	
Associates degree or higher	318 (53%)	279 (56%)	39 (37%)	
Income				
<USD 10,000	75 (13%)	58 (12%)	17 (17%)	<0.01
USD 10,000–29,000	175 (31%)	135 (29%)	40 (40%)	
USD 30,000–49,000	97 (17%)	82 (18%)	15 (15%)	
USD 50,000–69,000	69 (12%)	58 (12%)	11 (11%)	
USD 70,000–89,000	49 (9%)	44 (9%)	5 (5%)	
≥USD 90,000	102 (18%)	90 (19%)	12 (12%)	
Personal Cancer History, Yes	95 (16%)	75 (15%)	20 (19%)	0.36
Family History of Skin Cancer, Yes	202 (35%)	181 (37%)	21 (21%)	<0.01
Don’t Know (abs. cont.)	62 (10%)	43 (9%)	19 (18%)	<0.01
Absolute dichotomous, likely	191 (49%)	169 (52%)	22 (35%)	0.02
Don’t Know (abs. dich.)	207 (35%)	165 (33%)	42 (40%)	0.20
Test Offer	499 (83%)	406 (82%)	93 (88%)	0.17
Test Completion, acceptors ^1^	204 (41%)	194 (48%)	10 (11%)	<0.001
Risk Feedback, higher risk ^1^	73 (60%)	69 (60%)	4 (67%)	0.74
Skin Cancer Screening, Ever	223 (37%)	186 (38%)	37 (35%)	0.57

Note: *p*-values are based on Chi-square tests. Missing responses are as follows: race, *n* = 1; income, *n* = 33; ethnicity, *n* = 2; personal cancer history, *n* = 4; family skin cancer history, *n* = 16. ^1^ Test acceptance is only assessed for those offered testing (*n* = 499) and risk results are only assessed for those that accepted testing and received results before 3-month follow-up assessment (*n* = 114). Note: HS: High School; abs. cont.: absolute continuous; abs. dich.: absolute dichotomous.

**Table 2:** Patient continuous baseline characteristics by follow-up status (N = 600).

Mean (SD)	All, *n* (%)	Study Completers, *n* (%)	Lost-to-Follow-Up, *n* (%)	*p*-Value
Age	53.84 (14.3)	53.22 (14.0)	56.71 (15.3)	0.70
Burnability	0.48 (0.6)	0.47 (0.6)	0.53 (0.6)	0.43
Tannability	0.83 (0.6)	0.81 (0.5)	0.89 (0.6)	0.50
Lifetime Number of Sunburns	1.21 (1.2)	1.25 (1.2)	1.03 (1.3)	0.10
Health Literacy	10.59 (2.1)	10.73 (1.9)	9.92 (2.8)	0.19
Importance of Genetic Testing,	5.96 (1.5)	5.97 (1.5)	5.91 (1.7)	0.69
Perceived Risk				
Absolute continuous	3.95 (1.4)	4.00 (1.4)	3.71 (1.7)	0.31
Comparative cont.	2.87 (1.0)	2.90 (0.9)	2.72 (1.1)	0.79
Sun Protection	17.07 (3.8)	17.15 (3.8)	16.69 (4.0)	0.26
Skin Cancer Worry	2.54 (1.1)	2.53 (1.0)	2.58 (1.2)	0.68

Note: *p*-values are based on independent samples *t*-tests. Missing responses are as follows: lifetime sunburns, *n* = 8; importance of testing, *n* = 2.

**Table 3:** Model estimates (*p*-value) for test offer, completion, and receipt of risk feedback result, separately, on each study primary outcome (sun protection, skin cancer screening, and skin cancer worry) at 3 month follow-up.

Group	Model *n*	Sun Protection b (*p*)	Skin Cancer Screening OR (*p*)	Skin Cancer Worry b (*p*)
Test Offer (*n* = 406) vs. Usual Care (*n* = 87)	493	−0.12 (0.680)	1.24 (0.546)	−0.01 (0.873)
Baseline		0.68 (<0.001)	2.24 (0.002)	0.08 (0.001)
Complete (*n* = 194) vs. Decline Testing (*n* = 211)	405	0.36 (0.148)	0.97 (0.903)	−0.02 (0.765)
Baseline		0.68 (<0.001)	2.06 (0.013)	0.07 (0.008)
Higher (*n* = 69) vs. Avg (*n* = 45) risk feedback	114	0.35 (0.431)	0.82 (0.710)	−0.01 (0.921)
Baseline		0.71 (<0.001)	1.88 (0.230)	0.10 (0.045)

Note: Models regress the 3 month primary outcome (e.g., sun protection score) on the effect of interest (e.g., test offer) and control for the baseline score only. This table presents the results from 9 different generalized linear models, with an identity link used for sun protection score, logistic for skin exam, and Poisson for worry. Unstandardized regression coefficients (b) and odds ratios (OR) are presented.

**Table 4:** Effect sizes for moderator effects of risk results (higher vs. average) on sun protection, skin exam, and worry.

Moderator Variable	Sun Protection, η^2^ (*p*-Value)	Skin Exam, OR (*p*-Value)	Worry, η^2^ (*p*-Value)
Ethnicity	0.00 (0.67)	1.86 (0.62)	0.00 (0.97)
Race: White	0.00 (0.60)	1.03 (>0.99)	0.00 (0.96)
Gender	0.00 (0.50)	2.36 (0.55)	0.00 (0.94)
Education	0.00 (0.70)	8.32 (0.11)	0.01 (0.51)
Age	0.00 (0.80)	1.76 (0.39)	0.01 (0.68)
Income	0.00 (0.64)	2.29 (0.44)	0.00 (0.86)
Personal Cancer History	0.01 (0.19)	4.92 (0.18)	0.01 (0.50)
Family History of Skin Cancer	0.00 (0.62)	16.65 (0.05)	0.00 (0.79)
Burnability	0.01 (0.16)	1.34 (0.58)	0.01 (0.56)
Tannability	0.03 (0.01)	1.32 (0.59)	0.01 (0.65)
Lifetime number of sunburns	0.00 (0.93)	36.97 (0.01)	0.02 (0.41)
Health Literacy	0.00 (0.49)	1.46 (0.62)	0.00 (0.94)
Importance of Genetic Testing	0.00 (0.61)	2.97 (0.16)	0.00 (0.71)
Perceived Risk			
Absolute continuous	0.01 (0.09)	2.45 (0.13)	0.00 (0.89)
DK (abs. cont.): DK response	0.01 (0.09)	>99 (NaN)	0.00 (>0.99)
Absolute dichotomous: Likely	0.02 (0.04)	1.83 (0.63)	0.05 (0.20)
DK (abs. dich.): DK response	0.00 (0.96)	1.06 (0.96)	0.01 (0.48)
Comparative cont.	0.01 (0.11)	2.09 (0.20)	0.00 (0.99)

Note: Standardized effect sizes (η^2^ for continuous outcomes and odds ratio for the binary outcomes) and *p*-values are calculated for the interaction effect between the potential moderator and risk level (higher vs. average) on the given outcome. Each 3 month outcome is regressed on the baseline measure, risk level, the potential moderator, and the interaction term. Odds ratios (ORs) for continuous variables are standardized, representing odds ratios corresponding to an increase by one standard deviation of the distribution. Maximum n for each model is N = 114. Note: DK: don’t know; abs. cont.: absolute continuous; abs. dich.: absolute dichotomous

## Data Availability

The data presented in this study are available on request from the corresponding author.
